# Parents’ Experience and Views of Vaccinating Their Child against Influenza at Primary School and at the General Practice

**DOI:** 10.3390/ijerph15040622

**Published:** 2018-03-28

**Authors:** Pauline Paterson, Will Schulz, Martin Utley, Heidi J. Larson

**Affiliations:** 1 Department of Infectious Disease Epidemiology, London School of Hygiene & Tropical Medicine, Keppel Street, London WC1E 7HT, UK; william.schulz@lshtm.ac.uk (W.S.); heidi.larson@lshtm.ac.uk (H.J.L.); 2 Health Protection Research Unit in Immunisation, London School of Hygiene & Tropical Medicine, Keppel Street, London WC1E 7HT, UK; 3 Clinical Operational Research Unit, Department of Mathematics, University College London, 4 Taviton Street, London WC1H 0BT, UK; m.utley@ucl.ac.uk; 4 Department of Global Health, University of Washington, Seattle, WA 98104, USA

**Keywords:** vaccine acceptance, influenza vaccination, children, general practice, school vaccination, England, parental views, vaccination

## Abstract

The purpose of this study was to gain an in-depth understanding of parents’ experience and views of vaccinating their four to six-year-old child against influenza at school and at the general practice (GP). A cross-sectional qualitative study was conducted between March–June 2016 with parents of children in Reception and Year 1 in four randomly selected schools in Bury, Leicestershire, and Surrey, England. Twenty-five outreach forms were completed and returned, and seven interviews were conducted. Interview transcripts were coded by theme in NVivo (version 11, QSR International Pty Ltd., Melbourne, Australia). The primary reason parents gave for vaccinating their child was to prevent their child from contracting influenza. Parents’ perceived benefits of vaccinating in schools were to avoid the inconvenience of having to take their child to the GP, and that their child would behave better at school. Parents viewed that accompanying their child for the vaccination at school would undermine the convenience and peer-pressure advantages of the school as a venue. No parents expressed concern about their child being too young to be vaccinated in school. This research suggests that the school is a desirable venue for childhood influenza vaccination, both from the parents’ view and given that influenza vaccination coverage is higher when delivered through schools than GPs.

## 1. Introduction

In 2012, the Joint Committee on Vaccination and Immunisation (JCVI) recommended the extension of the influenza immunisation programme to children, based on an analysis which highlighted the cost-effectiveness of vaccinating children due to direct and indirect benefits to the individual and the population [1].

Due to the large population of children in England aged 2–17 years (~9 million) and to the large scale of the influenza programme, the programme is being implemented in phases [2]. The first phase started in 2013/2014, with 2 to 3-year-olds offered the influenza vaccine through general practices (GPs) and a pilot of 4 to 11-year-olds in seven geographical areas across England. In six geographical areas, the vaccine was offered through primary schools and in the seventh, a more rural area, the vaccine was offered through local pharmacies and GPs [3]. In 2014/2015, the nationwide programme was expanded from two to three-year-olds to additionally include four-year-olds (provided through GPs as before). The pilot with primary school children continued in seven geographical areas, and an additional 16 pilot areas introduced vaccination for secondary school students in Years 7 and 8.

In winter 2015/2016, all two to four-year-olds were continued to be offered the vaccine in GPs as part of the national programme [4]. In addition, the nationwide programme also included all children of school Years 1 and 2, through schools, GPs and pharmacies, depending on local commissioning arrangements. Four-year-olds who had started school could receive their vaccination either at a primary care service or at school depending on local commissioning arrangements [4]. The pilots continued in 4 to 11-year-olds in seven geographical areas.

Schools are an attractive venue for vaccination due to their ability to reach large numbers of children in short periods of time, and higher vaccination uptake has been shown in schools compared to primary care facilities and pharmacies [5,6,7].

In response to a request from the Department of Health, this study aimed to gain an in-depth understanding of parents’ experiences and views of vaccinating their four to six-year-old child against influenza at school and at the general practice (GP), during the 2015/2016 influenza season in England.

Study objectives:

- To explore parents’ understanding of the childhood seasonal influenza immunisation programme and possible benefits of participation;
- To explore parents’ experience with the seasonal influenza vaccination for their four to six-year-old child;
- To ascertain parents preferences on the vaccination programme;
- To understand parents’ decision-making process about whether to have their child vaccinated against seasonal influenza or not;
- To ascertain what would make children’s future participation in the influenza immunisation programme more acceptable to non-consenting parents.

## 2. Methods

### 2.1. Study Population, Recruitment, and Sampling

The study population consisted of parents and guardians of children in Reception Year and Year 1 in eight schools in Barnet, Bury, Leicestershire, and Surrey, England. These schools were randomly selected for participation in an evaluation by University College London (UCL) of the pilot implementation of the seasonal childhood influenza vaccination programme in England, during winter 2015/2016. The same schools were chosen in this follow up study.

Head-teachers and administrators at the pilot schools were contacted about the study in March 2016. Those that agreed to participate were provided with outreach packets to distribute to their Reception Year and Year 1 students to take home to their parents or guardians.

The outreach packets included a cover letter introducing the study, an information sheet giving further details about the study, and an outreach form (see Supplementary Material). The outreach form allowed respondents to register their interest in participating in an interview, and also included a brief set of questions about whether the respondent’s child had received the vaccine or not, and whether and why the respondent preferred vaccination at the school or the GP. The outreach packets included a pre-stamped and addressed envelope to return the outreach form to the investigators at the London School of Hygiene & Tropical Medicine (LSHTM).

Respondents who expressed interest in participating in an interview were contacted. An initial phone call was arranged for a researcher to further explain the study and answer any questions. If the respondent was still interested in participating in an interview, a time and place for the interview was arranged. 

Data was collected from both the completed outreach forms and through semi-structured interviews, conducted face-to-face or over the phone. Interviews followed a topic guide (see Supplementary Material) designed to probe participants’ general understanding and experience of the childhood seasonal influenza vaccination programme, their reasons for deciding to accept or refuse vaccination for their child, their understanding of the risks and benefits of this vaccine and vaccination in general, and their preferences and other factors that would make them more likely to agree to have their child vaccinated in the future. The interview topic guide was designed to encourage participants to give their views and opinions, and not with the intention of convincing them that they needed to vaccinate their child. Interviews were audio-recorded, with participants’ permission, and transcribed by a professional transcription service.

Of the eight schools approached, four schools agreed to distribute outreach packets to students. School A is in Surrey, Schools B and C are located in Leicestershire and School D is in Bury. In total, 398 outreach packs were distributed at these four schools. Of these, 25 outreach forms were completed and returned by respondents, of whom 12 expressed interest in an interview. Interviews were completed with seven participants in May–June 2016. The other five respondents were either unavailable or no longer interested in participating in an interview.

Outreach forms were received from three of the four schools that agreed to distribute outreach packets to their students. There were 16 outreach forms returned from School A (Surrey), only one from School B (Leicestershire), and eight from School C (Leicestershire). No responses were received from School D (Bury), which school administrators attributed to parents having difficulty understanding and answering the questions, due to limited English proficiency.

### 2.2. Data Analysis

Interview transcripts were coded with a thematic analysis technique, using the qualitative analysis software NVivo 11 (version 11, QSR International Pty Ltd., Melbourne, Australia). Analysis was conducted in tandem with data collection, and the first three transcripts were double-coded by two researchers (Will Schulz & Pauline Paterson), in order to develop an initial codebook with consensus around the key themes of the analysis. The data analysis was mainly thematic [8], although techniques outlined by Strauss and Corbin [9], which are common to grounded theory, were also be applied (i.e., open coding and the constant comparative method, allowing for the addition of new codes to accommodate additional themes emerging from subsequent interviews).

### 2.3. Ethics

This study was approved by the UCL Research Ethics Committee (Reference 5817/002) and the LSHTM Observational Research Ethics Committee (Reference: 10576). The study investigators obtained informed consent from participants and assured them that their anonymity would be maintained. Participants were informed that their participation was voluntary and that they were allowed to refuse to answer any question or end the interview at any time, without needing to give a reason for doing so. Recordings and transcripts of interviews were stored anonymously using a numerical identifier on password-protected computers. Only the investigators have access to the files that link a numerical identifier to a participant’s name.

## 3. Results

### 3.1. Descriptive Characteristics

Interview participants included five mothers and two fathers, with a mean age of approximately 39 years. In terms of ethnicity, four identified themselves as White British, one as White Other, one as White & Asian, and one as Indian. The religions represented included three Christians, one Hindu, one Atheist, and two participants with no religion.

**Figure 1:**
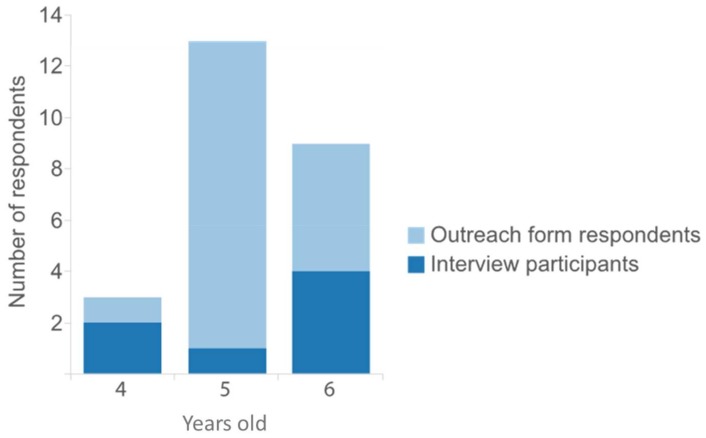
Respondents by age of child.

Figure 1 shows how response was distributed depending on the age of the child, with dark blue representing those who participated in an interview (in addition to returning the outreach form), and light blue representing those who returned the outreach form only. Parents and guardians of five-year-olds returned the greatest number of outreach forms (*n* = 13, 52%) compared with parents and guardians of six-year-olds (*n* = 9, 36%) and parents and guardians of four-year-olds (*n* = 3, 12%), but few of these respondents were both interested in and available for an interview.

Of the 25 outreach forms received, 11 parents indicated having vaccinated their child, and 14 indicated having not vaccinated their child against influenza. Of the seven participants interviewed, four had vaccinated their child against influenza, and three had not vaccinated their child. We focus here on three key themes which emerged from the interviews.

Themes resulting from the data analysis:

- Parents’ preference for vaccinating in school or at the GP
- Parents’ perceived benefits of vaccinating in school or at the GP
- Parents’ decision-making process about whether or not to have their child vaccinated against seasonal influenza
  ⚬ Reasons for accepting
  ⚬ Reasons for refusing

### 3.2. Venue for Vaccination: School versus GP

**Figure 2:**
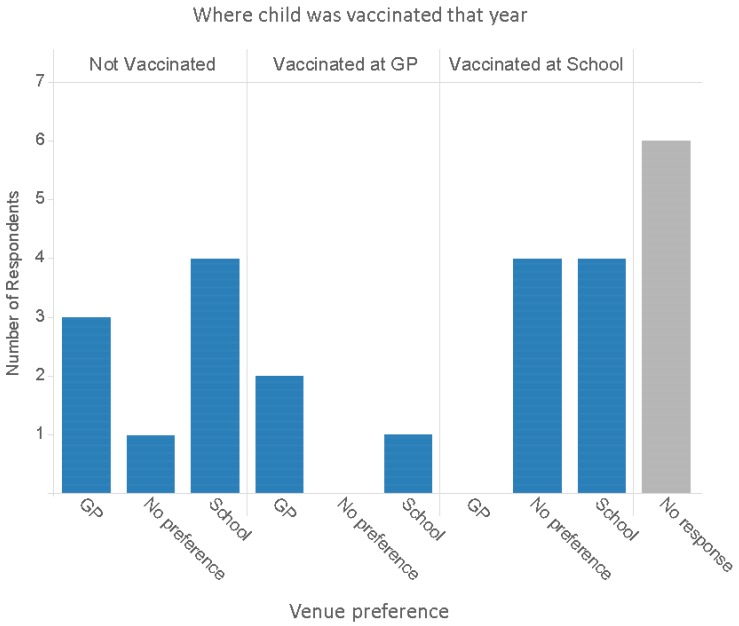
Venue preferences reported on the outreach form, disaggregated by location where child was vaccinated that year.

A key question this research sought to answer was whether parents prefer to have their child vaccinated at school or at the GP, and their reasons for this preference. Figure 2 gives an overview of venue preferences reported on the outreach form, broken down by whether their child was vaccinated at the GP, at school, or was not vaccinated at all. It is noteworthy that amongst respondents whose child was vaccinated in school, none expressed a preference for vaccinating at the GP in future. Parents whose child was vaccinated at the GP expressed interest in both venues (two expressed interest in vaccinating at the GP, and one in the school), as did parents whose child was not vaccinated against flu at all that year (three expressed interest in vaccinating at the GP, and four in the school).

A main perceived advantage of vaccinating in schools, from the parents’ point of view, was that it avoids the inconvenience of having to bring their child to the GP:

“It was inconvenient (at the GP). I work full time, I’m a single parent. So you know, I had to take time out to get them vaccinated… having it done at school is much easier.”

(P6 Interview, child vaccinated)

Some respondents had a negative experience with their GP’s availability and the process of booking appointments, which also links back to the issue of inconvenience noted above:

“Usually our GP is quite difficult. You ring up, you’re already like eighth in the queue and you’re waiting 20 min before you get through… then you have got to take your child out of school and get them there (to the GP).”

(P23 Interview, child vaccinated)

Four parents shared an insight relating to how children behave differently when they are with their parents at the GP, as opposed to when they are with their teachers and classmates at school:

“At school they all just go in and the child is not going to argue. They are not going to… children play to their parents as well. They know if they give you ‘Oh but mom’ you know you might soften up a little bit and just be like ‘Oh okay, not today’… In school they can’t give you their big sappy eyes and say ‘I want Mummy.’ And it’s just done.”

(P4 Interview, child vaccinated)

“At the GP you start now thinking, ‘Please don’t cry. Please don’t show me up, don’t scream the whole place down like you did last year’, and it’s quite a harrowing experience really.”

(P23 Interview, child vaccinated)

Moreover, parents expected their children to want to seem brave in front of their classmates, and saw this peer pressure as a way to make the vaccination go more smoothly:

“I would rather her done at school because her friends are having it done and it’s just less of a drama.”

(P6 Interview, child vaccinated)

“He is a little dude at school and he is big and brave in front of his friends, I think if they’re in a queue and they’re all together with their friends, they’d just get on with it…”

(P23 Interview, child vaccinated)

“They’re lemmings, literally sheep. They will all do what each other does… it’s quite regimental when you’re at school and it’s more of a confidence thing as well because your friends are doing it.”

(P4 Interview, child vaccinated)

Parents’ motivations for vaccinating at the GP, rather than at school, were less consistent. One respondent (whose child had an underlying health condition) was concerned that the school vaccination would not be given until the flu season was well underway. This parent nonetheless preferred vaccination at the school overall, but wished it had been conducted earlier in the year. Another respondent indicated on the outreach form that the GP was better because their son was autistic and would be less upset visiting the GP with a parent than if the vaccine were administered at school without the parent present. Several respondents said they preferred the GP because it was the normal or appropriate place to receive this sort of healthcare:

“Usual place to get any vaccination.”

(P21 Form, child not vaccinated)

“I feel more comfortable with it being done there (at the GP), I know them a lot better.”

(P17 Form, child not vaccinated)

“I will say GP, simply because this is the place where kids know they get this treatment or injections, and this is where (the) healing place is, and it might put kids off in school if you start giving vaccinations in school.”

(P12 Interview, child not vaccinated)

On the other hand, another participant made the exact opposite point about children’s association of pain with the GP:

“Plenty of times going to the GP can be a bit stressful, and you don’t want your child to start to associate pain with the GP visit. So you want, you know, that your children are comfortable in speaking to the doctor, for example, or do you want your child to be hiding from medicine if he’s feeling pain or something because… she doesn’t want to go to the GP.”

(P19 Interview, child vaccinated)

Considering school-based vaccination, no respondents expressed any concern about their child being too young to receive the vaccine in school. Moreover, when asked whether they would like to accompany their child for the vaccination in school, parents responded that this was an intuitively appealing idea, but impractical, and it would also undermine the convenience and peer-pressure advantages of the school as a venue:

“As a parent of course you (want to be there) because you sit at home thinking… ‘My poor baby’. But on the practical side... no. Because they are at school, they are doing what they ‘have to do’, and they can’t pull on anybody’s heart strings… I can imagine this little line of children… and then suddenly a parent is there and ‘Her parent’s there, mine’s not.’ Then that upsets them and they might get nervous and be like ‘I need my mom’.”

(P4 Interview, child vaccinated)

“The logistics around (having parents there) that is just a nightmare. You think if they are there with their peers then in theory they should just do it.”

(P5 Interview, child not vaccinated)

“I probably would have gone, whether that would have been the right thing to do or not; that was just sort of a mothering instinct really… However, whether that would have backfired on me I don’t know.”

(P23 Interview, child vaccinated)

### 3.3. Reasons for Accepting

The primary reason parents gave for consenting to have their child vaccinated with the influenza vaccine was to prevent their child from catching influenza, or from experiencing very severe symptoms:

“Well I did it for the benefit of my child, because I thought it would benefit my child health-wise. Also if there was the possibility that it would reduce the risk or the blow of full blown flu symptoms, then that is right up there on my list of important things.”

(P4 Interview, child vaccinated)

Parents also noted that ill children or teachers with influenza would need to miss school, and also that it can be disappointing if a family member had the flu and was therefore unable to celebrate the Christmas holidays.

“It can affect your family if… a teacher gets infected and cannot go to school or another kid gets infected… if people start not to vaccinate, that’s a problem… and actually it can spread into the population even if you are vaccinating people.”

(P19 Interview, child vaccinated)

“I know this sounds a bit selfish really, but you get to the Christmas holidays and then there’s usually some family that has been hit by flu and that’s Christmas ruined, and we’ve been there. I just think it would ruin a holiday time that we could have been enjoying our family time together and what not, it’s peace of mind that we’re not going to get it...”

(P23 Interview, child vaccinated)

Another motivation concerned the impact on GP practices:

“Doctors probably get inundated with people having flu… So if there is something that you can do to stop that epidemic of everybody bombarding the doctors that seems good to me.”

(P4 Interview, child vaccinated)

For some respondents, their doctor’s assurance allayed their concerns, particularly about the vaccine’s safety.

“The doctor reassured me that it’s all been tested and the side effects are minimal and there’s nothing to worry about. Having the Doctors reassurance was like crossing the ‘T’s and dotting the ‘I’s for me. I didn’t actually worry; that was all I needed really.”

(P23 Interview, child vaccinated)

“I think I trust the medical profession’s decision really. If they say it’s safe, then that’s good for me… I trust the medical profession to make up the decision as to ‘Yes this is better as opposed to not’.”

(P4 Interview, child vaccinated)

Some parents weighed up the risks and benefits of the vaccine:

“There is obviously a component of balancing risk and benefit. Depends on what kind of vaccination you’re offered too, but obviously it is clear that for some of the diseases you definitely want to be vaccinated.”

(P19 Interview, child vaccinated)

One participant received support from her doctor in weighing up the pros and cons of the vaccination:

“The first time [my child was offered the vaccine] I considered it for a long, long time and I looked it up… I took a few weeks; because obviously you’re wary, you hear horror stories and you don’t know what to trust really… I talked it through with the GP and once I had got her reassurance; I didn’t think about it. I just thought well okay, she knows what she’s doing. She actually did a bit of research for me because it was all quite new to her as well and she printed me off loads of stuff that she had got and sent it to me.”

(P23 Interview, child vaccinated)

For some parents, the cost–benefit calculation was strongly influenced by their child’s vulnerability to infection.

“Particularly in my daughter’s case I wouldn’t want her to get flu because she has got weakened lungs and things like that… she had actually been in HDU and the hospital advised that we vaccinate her and we vaccinated the whole family.”

(P6 Interview, child vaccinated)

“You are weighing it up is what you’re doing; and I’m thinking well, okay, she has got asthma which I have been told it could knock her more for six… and I hadn’t really heard of any side effects or anything happening.”

(P23 Interview, child vaccinated)

### 3.4. Reasons for Refusing

The most prevalent reason participants gave for refusing the influenza vaccine for their child was that their child was healthy, and not in a vulnerable group. Since only two participants who refused the vaccine agreed to an interview, we also include excerpts from the outreach form responses given by participants who refused the vaccine:

“She is generally in quite good health. I wasn’t sure if it was really necessary.”

(P5 Interview, child not vaccinated)

“She is a very healthy girl, who is very rarely poorly, so we chose not to vaccinate.”

(P16 Form, child not vaccinated)

“My children rarely get sick.”

(P18 Form, child not vaccinated)

“I think if we’d have had a child or somebody else in the house who was immuno-compromised … that would be different but we’re a healthy household, and it [having the vaccine] only seems to be a negative.”

(P7 Interview, child not vaccinated)

Despite forgoing the vaccine for her child, this same participant expressed interest in the idea that vaccinating her child could help protect the school community—similar to the parents who identified stopping the spread of flu as a reason for vaccinating—but in her case she did not feel that this rationale for vaccinating was promoted or supported by the informational materials she received:

“Yes, I wanted just more good arguments about why you would do it. If you don’t have a child that gets ill, it does not make any sense to have a flu vaccination, unless it’s like more reasoning say for the school, if it made a big difference to the school the fact that everybody had the vaccination, that would be interesting to know, but I felt that it wasn’t obvious and I couldn’t see any reason to do it.”

(P7 Interview, child not vaccinated)

The novelty of flu vaccination for children also contributed to the uncertainty about the purpose and benefits of this programme. One parent expressed a desire for more information about the impact of vaccinating:

“Because it sounded like something new I wasn’t 100% sure… even though it sounded good I wasn’t 100% sure… I wanted statistics and proof around so many percentage of children didn’t get the flu because of this. I think the thing is you don’t hear of many children actually having the flu. I think that’s the thing. If it was … not an epidemic, but if you knew it was as widespread as a cold or a sickness bug, there is obviously going to be more concrete evidence to prove it was a successful preventative. But because not many children get the flu I suppose it’s difficult to ascertain how successful it would have been. But it would have been nice to maybe find out some more statistics or information.”

(P5 Interview, child not vaccinated)

Several respondents (who declined an interview) returned outreach forms indicating broad doubts about the vaccine being unnatural or unsafe:

“I believe she should build up her own immunity naturally.”

(P25 form, child not vaccinated)

“Nutritional medicine is more effective and healthier than vaccines… Vaccines are unproven and can be toxic.”

(P18 form, child not vaccinated)

“Seen too many negative effects from others taking the vaccination.”

(P2 form, child not vaccinated)

While the latter response may be referring to the effects of the flu vaccine specifically, the first two clearly convey a broad distrust of vaccination in general, consistent with the views promoted by anti-vaccine advocates and “natural health” philosophies.

## 4. Discussion

### 4.1. Preferences about Vaccination Venue: School versus GP

A key finding is that, amongst respondents whose child was vaccinated in school, none expressed a preference for vaccinating at the GP in future. Also, no respondents expressed any concern about their child being too young to receive the vaccine in school.

While the practical convenience of in-school vaccination might have been expected to be its main advantage as is also seen in a U.S. study [10], parents also articulated the emotional challenges provoked by the GP setting, and being with the mother, which they considered to be attenuated in the school setting, where parents were not there and instead children looked to their peers. Children were perceived to express more anxiety about vaccination at the GP with their parents than at school with their classmates. However, it may be worth investigating whether children are feeling inward anxiety about having the vaccine at school without a parent present. If school-based vaccination is to be expanded in coming years this issue should be investigated, especially considering that existing literature suggests that early vaccination experiences may have consequences for subsequent vaccine hesitancy [11]. Furthermore, since existing research on school-based vaccination in the UK tends to focus on HPV vaccination in older (12 to 13-year-old) girls [12], the findings in other research may not be applicable to younger children, such as in the flu vaccination programme. Studies on school-based HPV programmes to date, are largely focused on girls, so additional research is also needed on boys, where relevant. 

A key public health benefit of vaccinating children at school, compared to primary care and pharmacies, is that higher vaccination uptake has been shown to be possible in schools [5,6,7,13].

### 4.2. Factors in the Vaccination Decision

Parents indicated that preventing their child from getting influenza was the primary purpose of vaccinating their child. Some parents readily embraced this rationale—especially those whose children had underlying health conditions that left them vulnerable to the flu. Other parents did not perceive influenza to be a major threat to their child’s health.

In 2012, the JCVI recommended the extension of the influenza immunisation programme to low risk children, based on an analysis which showed that vaccinating children with live attenuated influenza vaccine (LAIV) would be an effective and cost effective way to interrupt the transmission of influenza in the community at relatively low levels of uptake [1]. The childhood influenza vaccination pilots and programme only started in England in 2013/2014 flu season [14]. It is possible that parents assumed this new programme was only important for vulnerable children with special circumstances, which would explain the explanations from parents who refused the vaccine because their “child is healthy”. Alternatively, this response could reflect the view that if their child is healthy and has never had influenza, that they will not be spreading influenza to others, and so do not need to be vaccinated in order to protect others.

The promotional leaflet [15] for the 2015/2016 flu season, “Protecting your child against flu”, uses phrases such as “Flu can be a very unpleasant illness in children… Having the vaccine will help protect your child from what can be a very nasty illness”, which communicates that part of the reason to vaccinate your child is to protect them from the disease. Although in low risk groups, the case fatality is highest in 65+ year-olds, the highest incidence of hospital admissions for acute respiratory illness (ARI) is in the six-month to four-year-old age group [16]. When parents compared the costs and benefits of vaccination, several concluded that the risk of side-effects outweighed the benefits, when these benefits seemed unclear, or seemed to be relevant only to children with underlying health conditions. A US study also illustrated similar concerns about side effects [10].

The NHS leaflet states as the fifth reason to get your child vaccinated “protecting your child can stop flu spreading to other children and the family, especially babies and grandparents, who may be at higher risk from flu” [15]. Indeed, some participants supported the idea of vaccinating their child to protect their wider family and their school community, a personal desire to avoid the inconvenience of sick-days out of school, and the disappointment of sickness ruining holidays and family gatherings. Parents could be responsive to arguments that vaccinating their child may prevent disruptions to the school year and holiday arrangements, as well as protect the elderly and to reduce congestion at the GP. It may be worth reviewing the available evidence for benefits such as these, and including them as examples in future health promotion materials.

Some parents held a misconception that the vaccine would prevent other viral infections such as the common cold. It may be worth trying to correct this misconception, as they may incorrectly believe that the vaccine has been ineffective if a child then gets a common cold.

Finally, some interviewees communicated that they had great trust in doctors, and in at least one case a parent was convinced to accept the vaccine for her child because of her doctor’s efforts to seek out and share information about the vaccine. It has been well documented that a vaccine recommendation from a health care professional can increase the likelihood of vaccination [17]. It is also well-known that public trust in the medical profession can be leveraged in health promotion campaigns, for example by featuring real GPs in posters and billboards [18]. If it remains challenging to communicate the rationale for vaccinating children against influenza, then getting physician endorsements, or even personalising informational letters with a local GP’s signature, may increase confidence in this new programme.

This qualitative research gives in-depth insights into parent’s experience and views of vaccinating their 4–6-year-old child against influenza at school and at the general practice (GP), during the 2015/2016 influenza season in England. Given the small sample size of 7 interviews and 25 completed outreach forms, the findings are not generalizable, and cannot be extrapolated to be representative of all parents who were offered the flu vaccine at school in England. There is a possibility of sample bias, because those who responded to the outreach packet are likely to be more vocal and have stronger views on the subject than the average parent. In addition, this sample may under-represent people who were unaware of the vaccine offer, since those who did not receive the original notice of the vaccine offer would probably be more likely to also not receive the study outreach packet inviting them to participate in this research. However, the purpose of this qualitative research was to give an in-depth insight into the parental perspective, rather than provide a representative viewpoint.

## 5. Conclusions

The JCVI have recommended offering influenza vaccination through schools as the most effective route to deliver immunisations to school-aged children, since vaccination coverage levels in schools have been shown to be higher than in primary care and pharmacies. This research suggests that the school is a desirable venue to parents for influenza vaccination of their four to six-year-old children.

## Supplementary Materials

The following are available online at www.mdpi.com/1660-4601/15/4/622/s1, File 1: Cover letter, File 2: Topic guide, File 3: Outreach form, File 4: Information sheet.
