# Supplementary material for: Parents’ Experience and Views of Vaccinating Their Child against Influenza at Primary School and at the General Practice
Source: Int J Environ Res Public Health. 2018 Mar 28;15(4):622. doi: 10.3390/ijerph15040622 (PMC5923664; doi:10.3390/ijerph15040622)
Supplement: Supplementary file 1 [file ijerph-15-00622-s001.zip › Interview Topic guide for Parents and Guardians - flu attitudinal study v1 250116.docx]

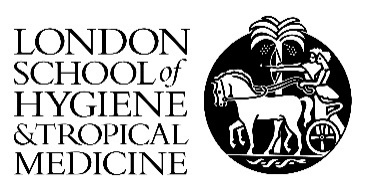
**
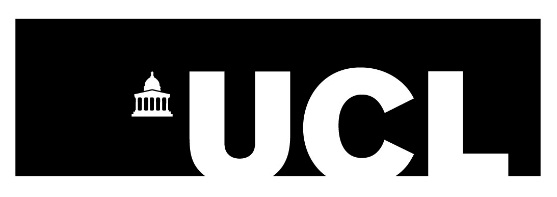
**

**INTERVIEW TOPIC GUIDE FOR PARENTS AND GUARDIANS**

**Parental views of the childhood seasonal influenza vaccination programme in England 2015-16**

Date of visit: _______________________ Place: ________________________________

Interviewer: ________________________________________________________________

**Interviewee(s) Socio-demographic characteristics**

Participant ID Number: _________________ Participant name: _________________________

Gender: Male Female Age (years):______________________

Ethnicity: ____________________________ Religion: _______________________________

Occupation: _______________________ Country of origin: ­­________________________

Relationship to child who was offered the vaccine: _____________________________________

Gender of child: ___________________ Age of child (years, months): _______________

School year of child: ________________ Location of vaccination (GP/school): _________

For children in year 1, location of vaccination last year (GP/school): _______________________

Number of members in household: __________

Other members of household: Relationship (no names to be recorded) and their ages (years)

_______________________________________________________________________________

_______________________________________________________________________________

_______________________________________________________________________________

*If the interview involves more than one person, add the details of the second person here:*

Name: _________________________________________________________________

Gender: Male Female Age (years):______________________

Ethnicity: ________________________ Religion: _______________________________

Occupation: ___________________ Country of origin: ­­________________________

Relationship to child who was offered the vaccine: _____________________________________

**Topic Guide**

*(Note – For children in Year 1, ask about this year and last year for relevant questions. For children in Reception, ask for this year)*

***Understanding and experience of the seasonal influenza vaccination programme***

- Tell us about your experience of your child being offered an influenza vaccine.
- What were your initial thoughts about the vaccination programme, and the possibility of your child taking part in this?
- What were you told about the programme and what information materials were you given? *(Probes – thoughts about information given, if you would have liked to receive other information)*
- What is your understanding of the reasons for the childhood seasonal influenza vaccination programme? *(Probes-purpose, benefits & risks)*
- What do you know about the vaccine Fluenz? (Probes – nasal spray, live vaccine)
- Has your child been vaccinated against influenza this year?
- Has your child been offered the vaccine in previous years? (Probes – If no, were you aware of the childhood flu programme? If yes, was your child vaccinated?)

If vaccinated

- What were your main reasons for vaccinating?
- Where was your child vaccinated? (Probes – School, GP, other)
- How did the vaccination take place? (Probes – If school, where in the school, were parents present? If GP – needed to book? Difficult to book? Difficult to find time to go? Had to take child out of school?)

If not vaccinated

- What were your main reasons for not vaccinating? (Probes – concerns of vaccine, lack of perceived need. If school, if parents are present or not. If GP, if need to take child out of school, difficult to book or find time to go)

***Decision-making about vaccinating their child against influenza***

- How did you make the decision about whether or not to vaccinate your child? *(Probes – what did this involve, weighing pros and cons, when did you make the decision and how long did it take)*
- What (if any) other information did you access to find out more about the vaccination programme or the vaccine? *(Probes – NHS/PHE websites, other websites, leaflets from other organisations)*
- Did you talk to others about deciding whether or not to have your child vaccinated? *(Probes – husband/wife/partner, other family members, friends, GP, religious leaders) (Note – do not collect names or any other personally identifiable data. Only record the relationship to the participant(s) and/or their professional roles)*

What were their opinions, and how did these influence your views?

- Who made the final decision whether or not to vaccinate your child?

***Risk-benefit considerations***

- What is your general view on immunisation*? (Probes – benefits, concerns, uptake of other vaccines)*
- What do you know about influenza? How serious do you think this is for children?
- What are the benefits of vaccinating your child against influenza?
- Have you or any of your close family members ever had influenza?
- Is anyone in your household offered an influenza vaccine because they are in a high risk group (e.g. diabetes, heart disease)?
- Have you or any of your close family members ever been vaccinated against influenza?

***Future vaccination***

- What would make you more likely to agree to have your child vaccinated? (Probes - convenient, access, safety/effectiveness explanations, more/different information about the vaccine and influenza, epidemic/perceived higher level of risk, household members susceptible to influenza, assurance from religious leaders)
- Would you be more likely to vaccinate your child if they were vaccinated at school or at the GP? *(Probes – reasons for preference)*
- If your child was being vaccinated at school, would you prefer to be there with them at the time? *(Probes – reasons for preference)*
- Do you think your preferences may change as your child gets older?
