# Supplementary material for: Parents’ Experience and Views of Vaccinating Their Child against Influenza at Primary School and at the General Practice
Source: Int J Environ Res Public Health. 2018 Mar 28;15(4):622. doi: 10.3390/ijerph15040622 (PMC5923664; doi:10.3390/ijerph15040622)
Supplement: Supplementary file 1 [file ijerph-15-00622-s001.zip › Study Information Sheet - flu attitudinal study v1 250116.docx]

**
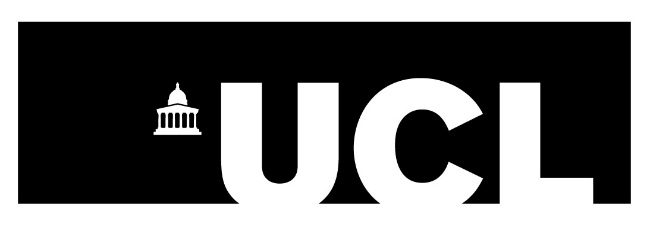
**
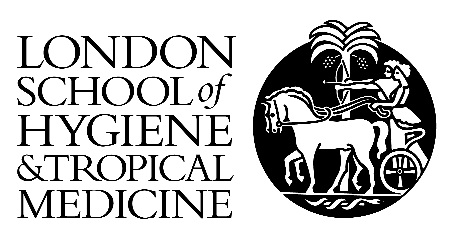


**Study Information Sheet - Parents and Guardians**

**Parental views of the childhood seasonal influenza vaccination programme**

You are being invited to take part in a research study. Before you decide whether you would like to participate or not, it is important for you to understand why the research is being done and what it will involve. Please take time to read the following information carefully and to talk to others about the study, if you wish. Ask us if there is anything that is not clear or if you would like more information. Take time to decide whether or not you wish to take part.

1. **What is the purpose of the study?**

The purpose of this study is to gain an in-depth understanding of how parents and guardians view the seasonal influenza vaccination programme for children, and their preferences as to where and how the vaccine is given, with the aim of informing the implementation of this programme.

**2. Why have I been chosen?**

Your child’s school was one of 12 schools that was randomly selected by UCL in 2014-15 to participate in an evaluation of the seasonal influenza vaccination programme. The areas selected include South-East Essex, South-West Essex, Havering, Leicestershire, Bury and South Tyneside. Children in Reception and Year 1 at these 12 schools are being invited to take part in our study. We would like to learn more about your views of the seasonal influenza vaccination programme, the reasons behind your decision whether or not to have your child vaccinated as part of this programme, and your preferences as to where and how the vaccine is given. In total we expect to interview 20-40 parents.

**3. Do I have to take part?**

It is up to you to decide if you would like to join the study or not and take part in an interview, and the information provided in this sheet should help you decide. If you are interested in taking part in the study please complete the expression of interest form and send this to us by using the freepost envelope provided. You can also contact us by leaving a message on Tel: 0207 958 8274 or sending an email to [schoolflupilot@lshtm.ac.uk](mailto:schoolflupilot@lshtm.ac.uk). Once you let us know that you are interested in taking part in our study a member of our research team will contact you. They will go through this information sheet with you and give you the opportunity to ask any questions you may have. If you agree to take part, we will arrange a time to either meet at your home or a place of your choosing or to have a phone call interview. Before you talk to us about your experience of the seasonal influenza vaccination programme you will be asked to sign a consent form. You are free to withdraw at any time, even during the interview, without giving a reason. Deciding not to take part in this study will not affect your relationship with your child’s school or your or your child’s access to health care.

**4. What will happen if I agree to take part?**

If you agree to take part in this study a researcher from the London School of Hygiene & Tropical Medicine will either visit you in your home or a place of your choice, or arrange a phone call interview, to talk to you about the childhood seasonal influenza vaccination programme. This will include talking about your views of the seasonal influenza vaccination programme, the reasons behind your decision whether or not to have your child vaccinated as part of this programme, and your preferences as to where and how the vaccine is given. Your views are of great interest and importance to us, and what you tell us will help inform the way the childhood seasonal influenza vaccination programme is conducted in future.

The interview will last about an hour and can be with one or both parents, or someone who is recognised as a legal guardian for the child who was offered a vaccine. The interviewer will take notes and with your permission the interview will be audiotaped. The audio-recordings from the interview will be transcribed into text, and anonymised so that the people taking part in the interview cannot be identified. We will store the interview data securely in line with Research Ethics Committee guidelines and only members of the research team will have access to this. We may use quotes from the interviews in reports and academic publications but these will be anonymous.

**5. Expenses and payments**

You should not incur any expenses from taking part in this study since the interviews will take place in your home or a place that is convenient for you, or we will be calling you if the interview is being carried out over the phone. To compensate you for your time and engagement we will provide you with a £20 Post Office multi-store gift card, which can be used at a wide range of shops.

**6. What are the possible disadvantages and risks of taking part?**

You may feel uncomfortable about talking about your decision regarding whether or not to have your child vaccinated against influenza, and your preferences as to where and how the programme is delivered. The researchers, who will be interviewing you, do not work for the NHS or any of the organisations who were involved in organising the seasonal influenza vaccination programme. They will respect your confidentiality and any information you share with them will be anonymised, which means that your names will not appear in any research documents.

**7. What are the possible benefits of taking part?**

Taking part in the study is unlikely to benefit you or your child directly, however the information you share with us will help inform the way that future childhood seasonal influenza vaccination programmes are organised.

**8. Will my taking part in the study be kept confidential?**

Yes. All information collected about you during the course of the research will be kept strictly confidential. Your name or your child’s name will not appear in any reports or publications and we will not tell anyone about your participation in this study.

**9. What will happen if I do not want to carry on with the study?**

You are free to withdraw from this study at any stage, even during the interview. If you want to withdraw from the study we will ask you whether you are happy for us to use any of the anonymous information you shared with us during the interview, or whether you would like us to destroy the recording and the transcript of your interview.

**10. What will happen to the results of the research study?**

The results of this study will be written up in a report which will be shared with people who are responsible for running and planning the childhood seasonal influenza vaccination programme. This includes staff who work for NHS England and related service providers, staff who work for Public Health England, and researchers based at the London School of Hygiene & Tropical Medicine and UCL. We will also publish findings from our research in academic journals and may comment on these on the London School of Hygiene & Tropical Medicine and UCL websites. We may be asked to comment on our research and findings by representatives of the media. You will not be identified in any report, publication or media communications and we will send you a summary of our research findings and a copy of the main published paper.

**11. Who is organising and funding the research?**

This research is being funded by the Department of Health, through UCL. This study has been outsourced to The Vaccine Confidence Project at the London School of Hygiene & Tropical Medicine. The principal investigator is Dr Pauline Paterson.

**12. Who has reviewed the study?**

This study was given ethical approval by the London School of Hygiene & Tropical Medicine Observational Research Ethics Committee and by UCL’s Research Ethics Committee.

**13. Contact Details**

If you would like to find out more or have any questions about this study please contact Pauline Paterson or Will Schulz on Tel: 0207 958 8274 or sending an email to [schoolflupilot@lshtm.ac.uk](mailto:schoolflupilot@lshtm.ac.uk). If you phone and there is not an answer, please leave a message on the answerphone, and we will get back to you as soon as possible. If you decide to take part in the study and subsequently have any concerns relating to your participation that you would like to discuss with somebody independent you can contact ethics@lshtm.ac.uk.

**Thank you for considering our study and taking the time to read this study information sheet.**
