# Supplementary material for: Parents’ Experience and Views of Vaccinating Their Child against Influenza at Primary School and at the General Practice
Source: Int J Environ Res Public Health. 2018 Mar 28;15(4):622. doi: 10.3390/ijerph15040622 (PMC5923664; doi:10.3390/ijerph15040622)
Supplement: Supplementary file 1 [file ijerph-15-00622-s001.zip › Cover letter - flu attitudinal study v1 250116.doc]

February 2016

Dear Parent / Guardian,

We are contacting you to hear about your views about the childhood seasonal influenza vaccination programme.

A study is being conducted by researchers from the London School of Hygiene & Tropical Medicine and the University College London (please see study information sheet enclosed). The main aim of this study is to learn about parents’ experiences of being offered the influenza vaccine for their 4-5 yr old child, including both those parents/guardians who chose to vaccinate their child and those who did not.

If you agree to take part in this study you will be asked to talk to a researcher about your views of the seasonal influenza vaccination programme, the reasons behind your decision whether or not to have your child vaccinated as part of this programme, and your preferences as to where and how the vaccine is given. Your views are very important and will help inform the way the seasonal influenza childhood vaccination programme is conducted in future. The researchers will respect your confidentiality and any information you share with them will be kept anonymous, and will not be used in connection with your name. Please note that participation is completely voluntary and deciding not to take part in this study will not affect your relationship with your child’s school or your or your child’s access to health care.

**We would be very grateful if you could complete the expression of interest form and return it to us using** **the enclosed freepost envelope.** We will then contact you to explain what the study involves and answer any questions you may have. If you would like to find out more about this study please do not hesitate to contact us.

Thank you for considering participating in this study.

Yours sincerely,

Will Schulz

Research Assistant

London School of Hygiene & Tropical Medicine

Email: [schoolflupilot@lshtm.ac.uk](mailto:schoolflupilot@lshtm.ac.uk)

Tel: 0207 958 8274
