# Supplementary material for: Parents’ Experience and Views of Vaccinating Their Child against Influenza at Primary School and at the General Practice
Source: Int J Environ Res Public Health. 2018 Mar 28;15(4):622. doi: 10.3390/ijerph15040622 (PMC5923664; doi:10.3390/ijerph15040622)
Supplement: Supplementary file 1 [file ijerph-15-00622-s001.zip › Outreach form - flu attitudinal study v1 260116.doc]

**Parental views of the childhood seasonal influenza vaccination programme:**

**Expression of Interest Form**

***Thank you for completing this form* ☺**

**1. Has your child been vaccinated against influenza this year?**

☐ Yes	☐ No

**What was your reason for this choice?**

______________________________________________________________________________________________________________________________________________________________________________________________________________________________________________________

**If yes, where was your child vaccinated?**

☐ School	☐ GP	☐ Other: ____________

**2. Where would you prefer to have your child vaccinated?**

☐ School	☐ GP	☐ Other: ____________	☐ No Preference

**What is your reason for this preference?**

__________________________________________________________________________________ __________________________________________________________________________________ __________________________________________________________________________________

**3. How old was your child on 1st September 2015?**

☐ 4yrs old	☐ 5yrs old	☐ other: ____________

**4. Would you be interested in taking part in this study?**

☐ Yes, I am interested in taking part in this study (please provide your contact details below)

☐ No, I do not want to take part in this study (no contact details required)

| **Name** |  |
| --- | --- |
| **Address** |  |
| **Phone number(s)**  **Preferred time to be called** | morning / afternoon / evening |
| **Email address** |  |
